# Exploring Olfactory–Oral Cross-Modal Interactions through Sensory and Chemical Characteristics of Italian Red Wines

**DOI:** 10.3390/foods9111530

**Published:** 2020-10-24

**Authors:** Elisabetta Pittari, Luigi Moio, Panagiotis Arapitsas, Andrea Curioni, Vincenzo Gerbi, Giuseppina Paola Parpinello, Maurizio Ugliano, Paola Piombino

**Affiliations:** 1 Department of Agricultural Sciences, Division of Vine and Wine Sciences, University of Naples Federico II, 83100 Avellino, Italy; elisabetta.pittari@unina.it (E.P.); moio@unina.it (L.M.); 2 Department of Food Quality and Nutrition, Research and Innovation Centre, Fondazione Edmund Mach (FEM), San Michele all’Adige, 38010 Trentino, Italy; panagiotis.arapitsas@fmach.it; 3 Department of Agronomy, Food, Natural Resources, Animals and Environment, University of Padova, 35020 Padova, Italy; andrea.curioni@unipd.it; 4 Department of Agricultural, Forest and Food Sciences, University of Turin, Grugliasco, 10095 Turin, Italy; vincenzo.gerbi@unito.it; 5 Department of Agricultural and Food Sciences, University of Bologna, 40126 Bologna, Italy; giusi.parpinello@unibo.it; 6 Department of Biotechnology, University of Verona, 37029 Cariano (VR), Italy; maurizio.ugliano@univr.it

**Keywords:** multi-modal interactions, red wine, astringency sub-qualities, taste, Volatile Organic Compounds (VOCs), deodorization, sensory analysis, chemical parameters

## Abstract

This work aimed at investigating red wine olfactory–oral cross-modal interactions, and at testing their impact on the correlations between sensory and chemical variables. Seventy-four Italian red whole wines (WWs) from 10 varieties, and corresponding deodorized wines (DWs), were evaluated by sensory descriptive assessment. Total phenols, proanthocyanidins, ethanol, reducing sugars, pH, titratable and volatile acidity were determined. PCA results highlighted different sensory features of the 10 wine types. ANOVAs (*p* < 0.05) showed that olfactory cues might play modulation effects on the perception of in-mouth sensations with 7 (harsh, unripe, dynamic, complex, surface smoothness, sweet, and bitter) out of 10 oral descriptors significantly affected by odours. Three weak but significant positive correlations (Pearson, *p* < 0.0001) were statistically found and supported in a cognitive dimension: spicy and complex; dehydrated fruits and drying; vegetal and unripe. In the absence of volatiles, correlation coefficients between sensory and chemical parameters mostly increased. Proanthocyanidins correlated well with drying and dynamic astringency, showing highest coefficients (r > 0.7) in absence of olfactory–oral interactions. Unripe astringency did not correlate with polyphenols supporting the idea that this sub-quality is a multisensory feeling greatly impacted by odorants. Results support the significance of cross-modal interactions during red wine tasting, confirming previous findings and adding new insights on astringency sub-qualities and their predictive parameters.

## 1. Introduction

Flavour results from the integration of all sensations perceived in the mouth and in the nose cavities, including olfactory (orthonasal and retronasal), tastes, and other oral sensations involving tactile and trigeminal perceptions [1,2]. During tasting, flavour perception is significantly affected by the interactions among sensory stimuli [3]. Wine is considered a hedonistic product and, more so today than in the past, its consumption and preference are sensitive to attributes such as quality, flavour and sensory characteristics [4,5]. Among all the sensory characteristics, odour and astringency are extremely important to define the complexity and quality of red wines and represent the two main intrinsic drivers of red wine consumers’ purchasing decisions [6,7,8].

Odour perception derives from the presence of Volatile Organic Compounds (VOCs). More than 800 volatile compounds have been identified in wines, with a concentration range varying from hundreds of mg/L to the μg/L or ng/L level [9]. However, only a few are present in concentrations above their sensory perception threshold [10] and involved in wine olfactory complexity. Astringency is a complex sensation mediated by both tactile and trigeminal receptors (mechanoreceptors and the trigeminal nerve located in the mouth) [11,12,13,14]. Although it is acknowledged that astringency stems from the interaction between tannins and flavan-3-ols with salivary proteins [15,16,17,18], the chemical and sensory aspects behind the elicitation of the different sensations ascribable to astringency and characterizing different red wine styles are still unknown. Due to its complexity, astringency has been described by 33 different terms, grouped in seven categories, among which two terms are frequently referred to smoother astringency characteristics (complex and surface smoothness), while the other five usually describe stronger stimuli (drying, harsh, unripe, dynamic and particulate) [19]. In the literature, the interactions between non-volatile and volatile wine fractions, and between the sensory stimuli elicited by their constituents, are broadly reported to influence wine chemical and sensory characteristics. Orthonasal and retronasal olfactory perceptions have been reported to be strongly influenced by wine chemical components, such as polyphenols, due to their effects on aroma release (see [20] and references therein). Astringency perception has been reported to be strongly influenced by wine chemical properties (i.e., pH, acidity, ethanol and polysaccharides) [21,22,23]. In the same way, some works addressed the study of multimodal interactions (i.e., aroma–aroma, aroma–taste, taste–astringency and aroma–astringency) and their sensory impact [24,25]. Notwithstanding the unclear mechanisms at the base of those interactions, it is known that they impact wine sensory perception and quality [26,27]. Hence, studying the cross-modal interactions of wine odour–mouthfeel stimuli is a subject of interest for wine researchers and producers to understand consumers’ perceptions and choices.

Cross-modal sensory interactions have been explored in model and real food products and beverages, such as cheese [28], cider [29], cocoa and milk beverages [30], desserts [31], olive oil [32], and yoghurt [33]. However, most of the studies present in the literature investigated aromas–mouthfeel sensations interactions in model matrices [34]. In the case of wines, a limited number of works focused on wine-like solutions, and, even fewer, on real wine matrices, showing, moreover, contradictory results. In an early study [35], applying a construction/deconstruction method, authors suggested that the addition of volatile fruity extracts from a Chardonnay white wine to the dearomatized non-volatile extracts of a red wine decreased astringency and bitterness and increased sweet perception. Vice versa, the substitution of a white wine volatile matrix with a red wine one, caused an increase in astringency perception and a decrease in sweetness. In a subsequent experiment [36], it has been demonstrated that the green mouthfeel character of red wines is positively correlated with vegetal aromas and negatively correlated with woody, ripe fruit and oxidized ones. Moreover, the relations between aromas and astringency has been further underlined [37], showing that in astringent model solutions with the presence of 2 g/L of catechin or epicatechin, the addition of specific volatile compounds with fruity, leather and smoked notes (due to isoamyl acetate, ethyl hexanoate, damascenone, 4-ethylphenol and 4-ethylguaiacol) increased the astringency persistence and intensity. Nonetheless, results from a very recent work [38], conducted with and without nose clips, reported that except for the oily mouthfeel attribute (which the authors hypothesised to be masked by earthy aromas and enhanced by alcoholic notes), the perception of aromas did not have an impact on the other palate sensations of red wines, including numerous astringency descriptors (i.e., dry, sticky, dusty, grainy, sandy, coarse, fleshy, mouthcoating, silky and gummy). Those results support a previous study [39]. Using a descriptive analysis technique based on intensity rating performed by three groups of participants (novices, trained and expert consumers), the study concluded that aroma–astringency interactions were quantitatively not relevant in determining the astringency intensity levels of red wines, regardless of consumers’ expertise level. By contrast, bitterness increased with animal aromas in the novice group. Therefore, the effect of aroma modulation on astringency and taste perception remains an unclear subject that needs to be explored further, ideally in real wines showing different sensory characteristics and matrix composition.

We recently studied the in-mouth sensory characteristics of 11 single-cultivar Italian red wines and we tested the correlations between sensory and chemical parameters [40]. The 11 wine types showed diverse astringency patterns characterized by a different balance among six astringency sub-qualities (drying, harsh, unripe, dynamic, complex, and surface smoothness). The correlations with compositional parameters were not tested considering the overall astringency as usually done in previous studies but by looking at its six sub-qualities and some chemical parameters, including total phenols and proanthocyanidins. Results partially support the hypothesis that olfactory cues related to wine VOCs might play a role in modulating the perception of some astringency sub-qualities. The exploration of this aspect is of interest as the research outputs are useful for oenologists to manage and control wine quality and to better comprehend consumer preferences/acceptance. Indeed, integrative brain processes, such as cross-modal interactions, could explain why it is difficult to find a direct correlation between specific compounds or chemical structures and astringency sensations that are of great interest for research and production.

For this reason, the main aims of this study were: (i) to investigate both odour–astringency (single sub-qualities) and odour–taste cross-modal sensory interactions in a wide set of real wine matrices, exploiting the sensory diversity of 10 single-cultivar Italian red wines; (ii) to test and compare the correlations between sensory (odour descriptors, astringency sub-qualities, and tastes) and chemical compositional parameters (total phenols, proanthocyanidins, ethanol, reducing sugars, pH, titratable acidity, volatile acidity) both in the presence and in the absence of VOCs. To do this, a sample set of 74 wines was assessed under two types of evaluation conditions: whole wines (WWs) and corresponding deodorized wines (DWs), meaning wines with or without odorants, respectively. In order to exclude olfactory perceptions, instead of using nose-clips as in most of the previous studies [24,38], a deodorization procedure was applied to make subjects comfortable with the sensory test and to simulate, as much as possible, the same breathing conditions experienced during a ‘normal’ wine tasting. Unlike previous methods applied for wine deodorization to study astringency or aroma [35,41,42,43], a new deodorization procedure was optimized to avoid the use of solvents and obtain representative deodorized wines that could be safely tasted by judges.

## 2. Materials and Methods

### 2.1. Chemicals

Fructose (99%) and tannic acid (95%) were purchased from J.T. Baker (Avantor; Radnor, PA, USA). Tartaric acid (99.7%) was provided by Chem-Lab (Eernegem, West-Vlaanderen, Belgium), and caffeine (99.2%) by ACEF (Piacenza, Italy). 2-Phenylethanol (≥99%), citral (95%), linalool (97%), 1-octen-3-one (96%), cis-3-hexen-1-ol (≥98%), ethyl butyrate (≥99%), damascenone (1.1–1.4 wt.%), benzaldehyde (≥99.5%), isoamyl acetate (≥95%), gamma-dodecalactone (≥97%), sotolone (≥97%), 4-ethylguaiacol (≥98%), 4-ethylphenol (99%), eucalyptol (99%), furaneol (≥98%), ethyl caproate (≥99%), eugenol (≥98%), citronellol (95%), phenylacetaldehyde (≥95%), furfuryl acetate (≥98%), 2,4,6-trichloroanisole (99%), 2-methyl-1-propanol (99.5%), methanethiol (≥98%) were all provided by Sigma-Aldrich (St. Louis, MO, USA). Ethanol (food grade, 70%) was supplied by ITW Reagents (Milano, Italy). Tanin VR colour, Tanin VR grape, Tanin plus, Tanin galalcool were all purchased from Laffort (Bordeaux, France).

### 2.2. Wine Samples

Seventy-four Italian red wines, 100% mono-varietal, vinified in 2016 from 10 Italian grape varieties harvested in 11 regions corresponding to the main geographical areas of production were sampled from the producers. The set of wines included: 10 Sangiovese (Romagna and Toscana), 8 Teroldego Rotaliano (Trentino-Alto Adige), 7 Corvina (Veneto), Raboso Piave (Veneto), Nebbiolo (Piemonte), Sagrantino (Umbria), Montepulciano (Abruzzo), Cannonau (Sardegna), Aglianico (Campania), and Primitivo (Puglia). Wines were selected from the most representative cellars of each production area, fermented in stainless steel vats at commercial scale, and sampled before malolactic fermentation and oak barrels ageing. Before bottling, all samples were protected with 50 mg/L of free SO_2_ before bottling, and bottles were closed with a Select Green 500 cork type (Nomacorc, Revisaltes, France) and stored at controlled cellar temperature (12 ± 2 °C) until analyses.

### 2.3. Sensory Analysis

The aim was to investigate the impact of the olfactory stimuli on astringency and taste perceptions during a red wine tasting. For this purpose, the 74 wine samples described above (whole wines: WWs) and corresponding 74 deodorized wines (DWs) were characterized for odour, astringency and taste features using a descriptive sensory assessment on a numerical category scale.

#### 2.3.1. Panel

The jury was composed of 14 selected individuals (7 males and 7 females aged between 22 and 49 years) recruited among students and researchers (Department of Agricultural Sciences, Division of Vine and Wine Sciences, University of Naples Federico II). They were selected based on their interest, availability, and ability to recognize olfactory and oral stimuli. They were all expert wine tasters with previous experience in performing sensory tests on wine. All procedures were conducted in accordance with the ethical standards of the institutional and/or national research committee and with the 1964 Helsinki declaration and its later amendments or comparable ethical standards. Participation was on a voluntary basis and, prior to the experiments, tasters were required to sign an informed consent form disclosing the type of research, voluntary participation and agreement to taste/smell reference solutions and wines. All data were collected anonymously.

#### 2.3.2. Procedure

Panel training: judges’ selection and familiarization with 10 in-mouth sensations (seven astringency sub-qualities: drying, harsh, unripe, dynamic, particulate/powder, complex, and surface smoothness/velvet; and three tastes: sweetness, sourness and bitterness) were performed according to the procedures and standard materials previously reported [40]. Likewise, panellists were selected and trained on olfactory stimuli by providing them a list of 11 odour families (fruity; dehydrated fruits; dried fruits: nuts; floral; vegetal; spicy; toasted; woody; earthy; alcoholic; off-odours: phenolic, sulphurous, cork taint, maderised/oxidised) selected from the literature [44] and 24 odour standards representative of different odour families and wine volatiles (Table 1).

Panellists were asked to smell each standard (20 mL of water solution in covered disposable plastic cups served according to a randomized order) to recognize the corresponding odour descriptor/s or family/ies and to score the intensity on the flowing numerical category scale: 1 = very low, 2 = low, 3 = medium, 4 = high, and 5 = very high, with half values allowed. One introductory session (no data collected) and two real sessions were carried out. Data collected in the 2nd and 3th sessions were used to calculate the frequency of citations for standard correctly matched with descriptor/s. Only the terms with an association frequency (percentage of judges that consensually matched the correct descriptor to a given standard solution) ≥85% were considered as consensually associated to the corresponding standards (Table 1). At the end of each training session, the perceived sensations were discussed with the participants to prevent overlapping and redundancies among terms and to help their memorization.

Finally, in order to familiarize the jury with the application of the procedure to real wines as well as to test panellists’ performances, 10 commercial wines (selected among samples under investigation) were assessed (two replicates) using the same evaluation procedure as run-through prior to the real analytical sessions. Subjects were provided with water and required to wait at least 15 s between each sample.

Sensory assessment: WWs and DWs were analysed by descriptive sensory assessment using the same vocabulary and the nine-point numerical category scale employed during the training. A total of 296 samples, meaning (74 WWs + 74 DWs) × two replicates, were assessed during 15 sessions (10 wines/session; the two missing wines were obtained by blending some available wines but data on these “fake” samples were not considered in the analyses). Each session was split into 2 sub-sessions with an imposed break of 15 min, and the evaluations of WWs or corresponding DWs were performed in each sub-session. All participants evaluated the 74 WWs by first smelling and scoring odours intensities, and then by tasting for astringency sub-qualities and tastes according to the procedure previously described [40]. The same tasting procedure was repeated on DWs in a separated sub-session. Subjects were not informed about the nature of the samples. For each sample, 25 mL were served in covered glasses [45] coded with three-digits and presented in a randomized order. Wines were served at room temperature (21 ± 1 °C) and evaluated in individual booths [46].

### 2.4. Deodorization and Reconstitution of Wines

Drawing from the methods previously reported [43,47], a new rapid (~2 h) deodorization procedure was optimized to obtain representative and safe deodorized wines (DWs). The whole wines (WWs) were deodorized during the two days preceding the date fixed for the corresponding session of assessment. Wines were deodorized one by one (two replicates) as follows: 360 mL of wine were weighed and treated in an ultrasound bath (Transsonic 460 H, Elma, Germany) with water as processing liquid, working at a fixed frequency of 35 KHz, to the minimum intensity (1) in a range between 1–15 (set through a turning knob), and maintained at a controlled low temperature of 20 °C for 30 min. The samples were then evaporated at 30 °C under reduced pressure (Rotavapor R-210, Büchi, Switzerland). The process was stopped when the samples reached a weight loss of ~95% (~90 min). As the deodorization procedure stopped, the samples were weighed and reconstituted, one by one, at the initial weight by adding distilled water and food-grade ethanol at a proper concentration to reach the initial alcohol degree (%*v*/*v*) of the wine. DWs were then stored at (12 ± 2 °C) till the analysis. Any visual differences between reconstituted wine and real wine were ascertained on a subset of samples randomly chosen within each grape variety, by means of discriminant analysis (triangle test, [48]): differences resulted not significant (α = 0.01). This test, along with an informal check to verify the absence of off-odours and off-tastes, was conducted internally at the laboratory. The efficacy of the deodorization was confirmed by Gas-Cromatography/Mass Spectrometry (GC-MS) analysis [49] of the volatile fraction of the wines prior and after deodorization–reconstitution. Different methods for VOCs isolation were applied for the check: pre-concentration by SPME (Figure S1) and liquid–liquid extraction as previously reported [50,51].

### 2.5. Chemical Analysis

Ethanol, reducing sugars, volatile acidity (VA), and titratable acidity (TA) were measured according to the Organisation Internationale de la Vigne et du Vin (OIV) methods [52]. pH was determined by potentiometry (InoLab 730 pH meter, WTW, Weilheim in Oberbayern, Germany). Total phenols were measured by Folin–Ciocalteu assay [53]. The concentration of proanthocyanidins was determined after acid hydrolysis with warming (Bate-Smith reaction) using a ferrous salt (FeSO_4_) as catalyst [54,55]. Analyses were carried out in triplicate.

### 2.6. Data Analysis

For the sensory characterization of WWs, two Principal Component Analyses (PCA) were carried out on the correlation matrices (Pearson, *p* < 0.05) of the mean intensities over wines of each grape variety rated by the 14 judges for significant in-mouth sensations and odours, and tested using multi-way ANOVAs.

A three-way ANOVA (judges as random factor, grape variety and perception modality as fixed factors; Tukey, *p* < 0.05) with interactions (grape variety*perception modality) was computed to test the discrimination effect of in-mouth descriptors and to evaluate the impact of the perception modality (with and without VOCs, WWs and DWs respectively) on astringency sub-qualities and tastes perception across the 74 red wines under investigation. A two-way ANOVA (judges as random factor and wine variety as fixed factor; Tukey, *p* < 0.05) was also computed to test the discrimination effect of olfactory descriptors across the 74 wine samples. To test the impact of olfactory cues on the perception of the astringency sub-qualities in the 10 mono-varietal wines, other two-way ANOVAs (judges as random factor and wine as fixed factor; Tukey, *p* < 0.05 and 0.1) were performed on the intensity scores of astringency sub-qualities in WWs and corresponding DWs of each wine type.

Pearson correlation analyses (*p* < 0.05) were applied to the whole set of wines (sample size: 74) for the computation of correlations between specific odour descriptors and in-mouth sensory variables, and between these latter for WWs or DWs and chemical parameters.

Performance of the trained judges was tested by a three-way ANOVA (Tukey, *p* < 0.05) with three interactions: assessor*session, assessor*sample, sample*session [56].

Data was processed with XLStat (version 2019.6), an add-in software package for Microsoft EXCEL (Addinsoft, Paris, France).

## 3. Results and Discussion

### 3.1. Olfactory/in-Mouth Cross-Modal Interactions

**Figure 1:**
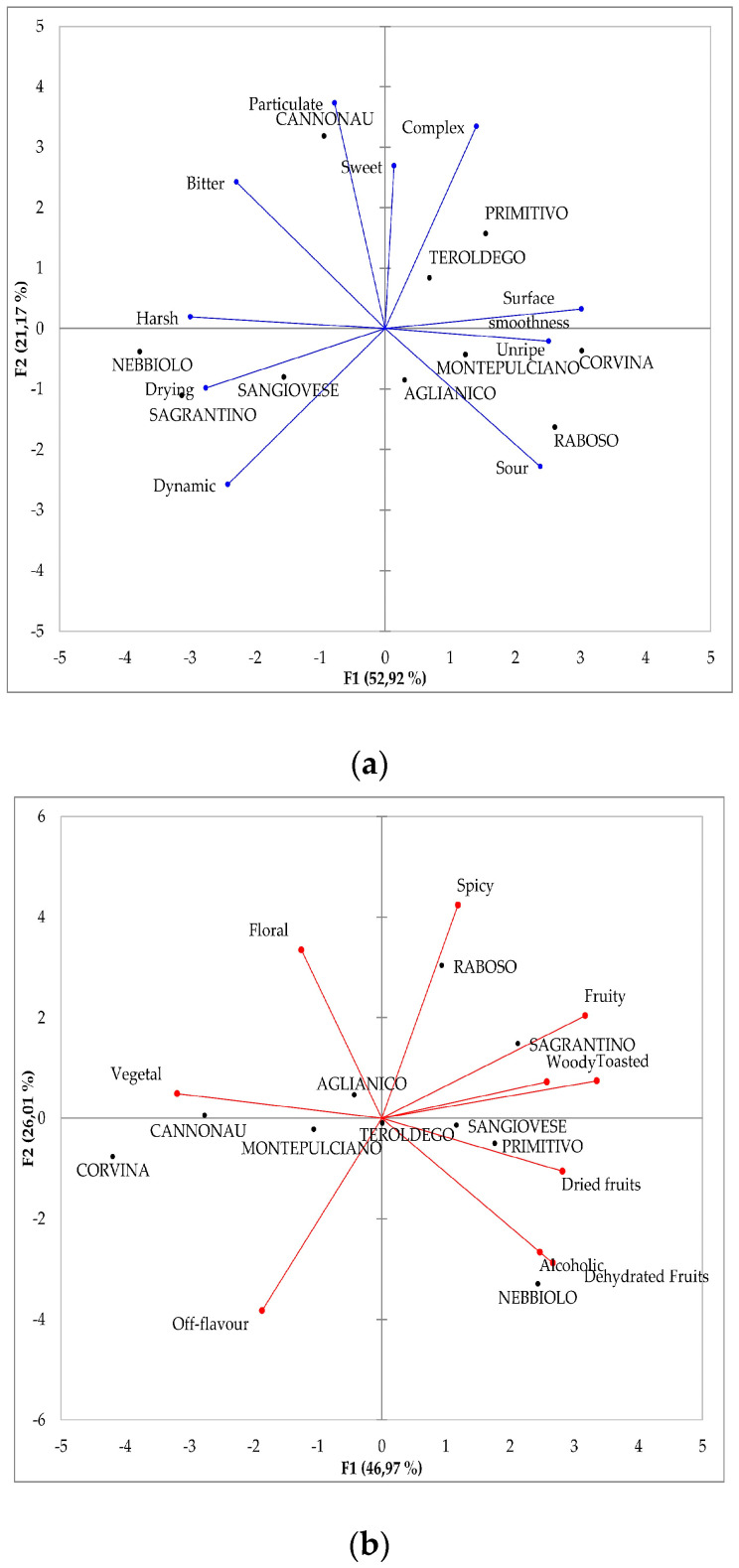
Principal component analysis (PCA) plots carried out on the correlation matrices (Pearson, *p* < 0.05) of the mean intensities over the 10 single-varietal wines rated by the 14 judges for significant: (**a**) oral (astringency and tastes) characteristics and (**b**) olfactory attributes.

The main aim of this study was to investigate the impact of olfactory cues on tastes and astringency sub-qualities during red wine tasting. In order to account for the wide sensory diversity that different red wines can show, the experiments were carried out on 74 wines selected among the 111 Italian red wines (11 grape varieties), whose astringency has been recently studied [40]. As a first step, we tested the sensory diversity of the 74 wines produced with 10 grape varieties. As a result, the discrimination effect of oral and olfactory descriptors among the 74 wines was tested by ANOVA and results are reported in Table 2 and Table 3, respectively. The sensory features of the 10 single-varietal wines are shown in two separated PCAs computed on the mean intensities of oral (astringency and taste) characteristics (Figure 1a) and olfactory attributes (Figure 1b), respectively. The first biplot (Figure 1a) accounts for more than 74% of the variance, while the second (Figure 1b) for around 73%. The charts show the sensory diversity of the 10 wine types.

In Figure 1a, Corvina and Raboso show the largest squared cosines to positive values of F1, where the variables surface smoothness, unripe and sour taste are well projected. Montepulciano and Aglianico occupy the same area but show lower squared cosines. On the opposite side of F1, Nebbiolo, Sagrantino and Sangiovese are all well correlated to harsh, drying and dynamic astringency. Particulate, complex and sweet sensations are well represented on positive F2 and correlated with Cannonau, while Primitivo and Teroldego are mostly correlated with complex and smooth astringency. Figure 1b shows that the sample set was representative of wines with different olfactory characteristics. F1 represents the contrast between wines with dominant vegetal odours, mainly Corvina and Cannonau, and those presenting different notes: fruity, toasted and woody odours such as Sagrantino; dried fruits like Primitivo; dehydrated fruits and alcoholic notes like Nebbiolo. On F2, opposite to off-flavours, there are wines with spicy and floral odours, such as Raboso and Aglianico, respectively. This latter wine along with Montepulciano, Sangiovese and Teroldego, has low squared cosines suggesting a lower and/or more balanced contribution of different odours.

Except for particulate/powder astringency, all the other nine in-mouth descriptors showed significant effects for the fixed factor grape variety (Table 2). Eight out of 11 olfactory descriptors resulted significantly different depending on the grape variety (Table 3): dried fruits (nuts) and woody were not significant and so it was the earthy descriptor, which was not considered for further analyses—including the PCA reported in Figure 1b—due to the lack of significance of its model.

These first results confirm an inter-varietal sensory diversity of the 10 monovarietal wines. This diversity represents the assumption for the investigation of cross-modal sensory interactions between olfactory cues and in-mouth sensations during red wine tasting.

**Figure 2:**
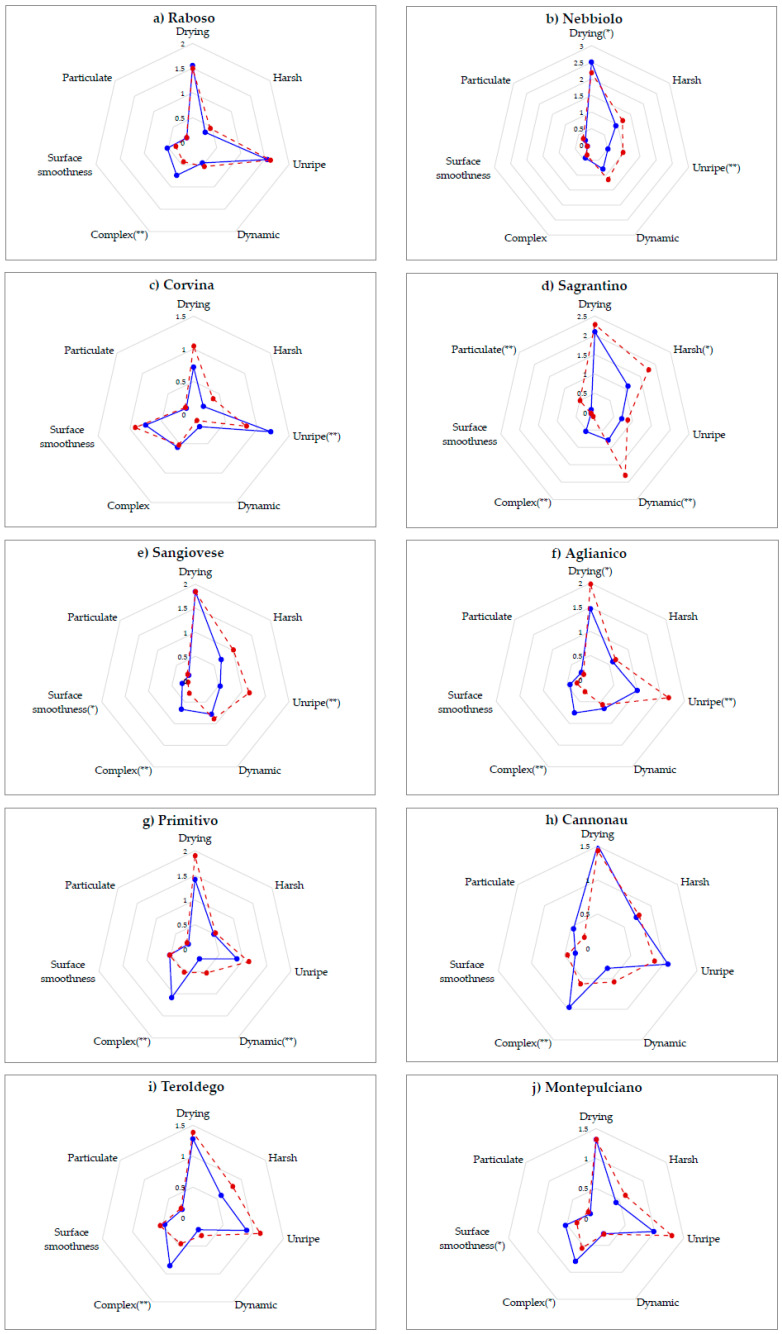
Spider-plots illustrating the impact of the deodorization on astringency sub-quality profile of each of the 10 single-varietal wines: (**a**) Raboso, (**b**) Nebbiolo, (**c**) Corvina, (**d**) Sagrantino, (**e**) Sangiovese, (**f**) Aglianico, (**g**) Primitivo, (**h**) Cannonau, (**i**) Teroldego and (**j**) Montepulciano. Significant differences assessed in WWs (blue line) and corresponding DWs (broken red line) are marked with asterisks (* *p* < 0.1, ** *p* < 0.05).

The spider-plots in Figure 2 illustrate how the astringency sensory profile of each of the 10 single-varietal wines changed after deodorization. We worked under the assumption of representative deodorized samples not only because our procedure has been developed from previous ones [43,47]. We also considered that rotary evaporation at low temperature (30 °C) represents a method largely applied during the preparative steps for polyphenols analysis in several food matrices (including wine) by several methods [57]. Based on the actual literature [58,59,60,61,62,63], possible chemical, physical chemical and rheological changes of polyphenols due to ultrasound and/or evaporation treatments are not favoured under the working conditions applied and, moreover, there is no clear evidence of their significant and/or irreversible impact on the sensory characteristics of the wine. As an example, the reported maximum effects of ultrasound treatment on the chromatic characteristics of wine is ΔE* = 2.8 [61]. Considering that the theoretical limit of perception reported for the human eye is ΔE* ≥ 3 [59], this means that differences cannot be detected from a sensory point of view. This is coherent with our results from triangle test showing that the colour of WWs and corresponding DWs were not perceived as different. Moreover, a recent study on the application of ultrasounds to accelerate the autolytic process in wine yeast [60], did not detect any effect on sensory parameters, including colour intensity, tonality, body, astringency, acidity, global quality and bitterness.

The ANOVA highlights several significant differences (*p* < 0.05, *p* < 0.1) in mean intensities of perceived sub-qualities assessed in WWs and corresponding DWs. At least one significant variation resulted for each wine type. Sagrantino’s astringency was impacted the most after deodorization, with four astringency sub-qualities (harsh, dynamic, complex and particulate) whose mean intensity significantly varied in the absence of olfactory cues. Three significant variations were detected for both Sangiovese (unripe, complex and surface smoothness) and Aglianico (unripe, complex and drying) and two for Nebbiolo (unripe and drying) and Primitivo (dynamic and complex). The astringency of the remaining wines was less affected by the absence of VOCs, where significant variations were detected only for one sub-quality, namely complex for Raboso, Cannonau and Teroldego, and unripe for Corvina.

Two sub-qualities were the most frequently impacted by the deodorization: complex was perceived as significantly less intense in 8 out of 10 wine types (Raboso, Sangrantino, Sangiovese, Aglianico, Primitivo, Cannonau, Teroldego and Montepulciano) and unripe in four (Nebbiolo, Corvina, Sangiovese and Aglianico). This is not surprising because both these astringency sub-qualities correspond to sensations including not only oral but also retronasal olfactory perceptions. Indeed, based on the original definitions [19], our jury developed and used consensual definitions as previously reported [40]: complex was intended as a balanced in-mouth sensation of smooth astringency, acidity and retronasal stimulation; unripe corresponded to an unbalanced in-mouth sensation of astringency, acidity and green aroma.

The direction of the variation was always the same for all sub-qualities across all monovarietal wines, except for two terms: drying that slightly varied (*p* < 0.1) in Nebbiolo and Aglianico but in opposite direction; and unripe that increased (*p* < 0.05) for deodorized Nebbiolo, Sangiovese and Aglianico, while decreased (*p* < 0.05) for deodorized Corvina. This result could be linked to the strong vegetal odours detected in these wines (Figure 1b), in line with previously reported high concentration of VOCs, such as cyclic terpenes and hexanols, characteristic of Corvina wines and responsible for its vegetal/herbaceous/balsamic character [64,65].

A recent study [36], aimed to identify chemical compounds driving green character in red wines, concluded that it is a multivariate character associated to both aroma and mouthfeel descriptors such as vegetal, astringency, green and dry tannins. Based on this knowledge, our hypothesis is that the strong vegetal odours of Corvina can enhance the perception of unripe astringency. This synergic/additive effect could be the reason why, unlike Nebbiolo, Sangiovese and Aglianico (Figure 2b,e,f) that were not characterized by vegetal odours (Figure 1b), in Corvina the unripe astringency was perceived more intense in WWs (Figure 2c). This hypothesis seems to be supported by a similar trend detected in Cannonau (Figure 2h) which, like Corvina, was strongly characterized by vegetal odours (Figure 1b).

In order to get a more general result, an ANOVA (*p* < 0.05) was applied across the whole set of 74 wines belonging to the 10 different grape varieties, to evaluate the impact of the perception modality (presence or absence of VOCs) and of the interaction “perception modality*grape variety” on in-mouth sensations assessed in WWs and DWs.

**Figure 3:**
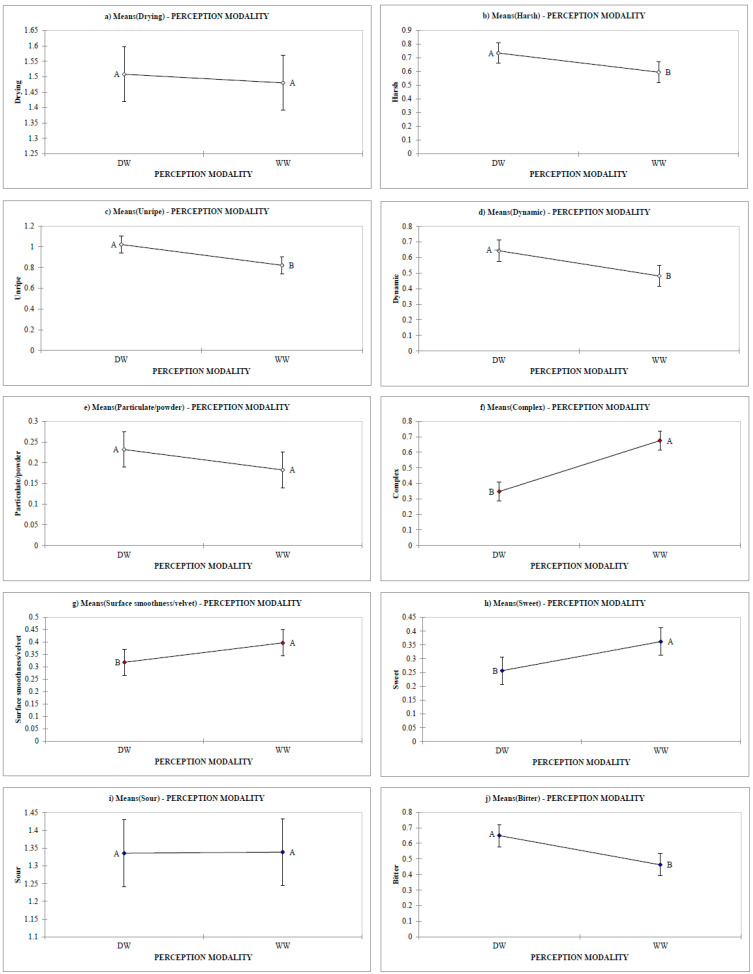
Variation of mean intensities (over 74 wines) of each astringency sub-quality and taste sensation during DWs tasting compared to corresponding WW: (**a**) Drying, (**b**) Harsh, (**c**) Unripe, (**d**) Dynamic, (**e**) Particulate/powder, (**f**) Complex, (**g**) Surface smoothness/Velvet, (**h**) Sweet, (**i**) Sour and (**j**) Bitter. Significant differences are marked with different letters (*p* < 0.05). whole wines (WWs), deodorized wines (DWs).

Results reported in Table 2 show that the perception of 7 (harsh, unripe, dynamic, complex, surface smoothness, sweet and bitter) out of 10 in-mouth sensations was significantly affected by odours. Complex astringency is the most impacted by olfactory cues (*p* < 0.0001) while both the unripe and dynamic sub-qualities were significantly affected by the interaction “perception modality*grape variety”. The variation of mean intensities (over 74 wines) of each astringency sub-quality and taste sensation during DWs tasting compared to corresponding WWs is represented in Figure 3.

Except for drying and particulate (Figure 3a,e), the other astringency sub-qualities (harsh, unripe, dynamic; Figure 3b–d) were perceived stronger in DWs. This suggests that olfactory perception can smooth these mouthfeel sensations previously described as “strong astringency sensations” [40,66]. On the contrary, complex and surface smoothness/velvet (Figure 3f,g) decreased in DWs, suggesting that olfactory cues can enhance smoother aspects of astringency. The lack of impact on particulate astringency could be because wines were not discriminable according to this astringency feature. The perception of drying astringency that, based on results from consumer studies [67], is assumed to be the basic astringent sensation because the most easily associated to the global term astringency, was not significantly impacted by olfactory cues. A similar result has been already reported [38].

Moving to taste sensations (Figure 3h–j), it can be observed that the perception of olfactory stimuli significantly impacted bitterness and sweetness. Bitterness increased in the absence of VOCs, in accordance with previous data [38], whereas perceived sweetness decreased. Those results seem to confirm the ones of an earlier study [24], in which wine sweetness and bitterness perceptions were found to be significantly impacted by aromas. Moreover, previous findings on the effect of aromas on cider tastes showed that, overall, aromas significantly modulated sweetness perception for ciders with a sugar content of around 35–40 g/L [29]. Although the residual sugar content of our samples was 1 to 20 g/L (Table 4), our results are in line with the mentioned work. Sourness sensation did not show significant differences between WWs and DWs, meaning that the perception of olfactory stimuli did not influence this taste attribute.

According to Figure 1b, the large set of wines showed a wide array of sensory characteristics matching the large range of basic compositional data reported in Table 4. Thanks to this diversity, we tried to go deeper into our investigation on cross-modal interactions in red wine tasting, by performing a Pearson correlation analysis to statistically test the relationships between specific olfactory notes and single astringency sub-qualities and tastes. Results report a total of 21 significant (*p* < 0.05) correlations, 17 significant correlations between odours and astringency sub-qualities and 4 between odours and tastes. However, in most cases the computed r value is very low and likely linked to a casual effect. For each astringency sub-quality, one to four significant correlations to olfactory descriptors were found. Fruity was slightly correlated with the complex astringency (r = 0.308). In a previous study [35], it was observed that the addition of a fruity aroma extract coming from a Chardonnay white wine caused a significant decrease in the perception of the global astringency in different red wine matrices. Lately, this output was not confirmed [38]. The descriptor dehydrated fruit positively correlated with drying (r = 0.459; *p* < 0.0001) and harsh astringency (r = 0.286), while negatively correlated with surface smoothness (r = −0.341). This could suggest that these three sub-qualities are parts of one unique sensation, where smoothness complements strong sensations such as drying and harsh. A similar consideration was recently reported for silky and dry mouthfeel descriptors [38]. Dried fruit was the only odour descriptor never correlated with in-mouth sensory variables. Floral aromas showed very weak relationships: positive with the complex sensation (r = 0.275) and negative with harsh (r = −0.284). Vegetal odours were the only ones related to four sub-qualities. The correlation with unripe (r = 0.385; *p* < 0.0001) and surface smoothness (r = 0.237) astringency was positive while the correlation with drying (r = −0.340) and dynamic (r = −0.291) was negative. Spicy only correlated with complex (r = 0.462; *p* < 0.0001) but it had the largest coefficient both within the whole dataset and, compared to the other odours correlated to this sub-quality: fruity and floral positively and off-flavour (r = −0.307) negatively.

These relationships are based only on a statistical approach and, as already stated, the low r values, suggest a casual effect. However, the three largest and significant correlations (*p* < 0.0001) that were found—spicy and complex, dehydrated fruits and drying, vegetal and unripe—seems to be confirmed from a cognitive point of view. Indeed, according to Figure 1b, Raboso were the spiciest wines and after deodorization their astringency was perceived as significantly (*p* < 0.05) less complex (Figure 2a), confirming the significant and positive correlation previously reported (r = 0.462). Nebbiolo was characterized by dehydrated fruits odours (Figure 1b) and the average astringency of deodorized Nebbiolo was perceived as less drying (*p* < 0.1), in line with the computed positive correlation (r = 0.459). Finally, in accordance with the positive significant correlation (r = 0.385) between vegetal odours and unripe astringency, in Corvina wines, which were strongly characterized by vegetal notes (Figure 1b), the unripe astringency was perceived significantly (*p* < 0.05) less intense in DWs compared to WWs (Figure 2c). A similar finding (even if not significant) was observed for Cannonau, which was the only other monovarietal wine associated with vegetal odours (Figure 1b). The green character has been negatively correlated to consumers’ preference of red wines, resulting in intensified vegetal notes and masked by woody aromas [36]. Our results support both these conclusions: woody odours were significantly (*p* < 0.05) correlated with unripe astringency, even if with a small negative correlation coefficient (r = −0.257). Moreover, alcoholic notes were negatively correlated (r = −0.340) with unripe astringency. These results are interesting and need to be verified by further experiments.

Few significant correlations were detected between olfactory characteristics and taste sensations and, also in this case, the r values were very low. Sweet taste did not correlate to any odour, while sourness was positively correlated to floral and bitterness showed a low negative correlation with floral and a positive correlation with the dehydrated fruits and off-flavour. This latter descriptor was intended as inclusive of different kinds of wine off-odours (phenolic, sulphurous, cork taint, maderised/oxidised); however, the most cited off-odour was the phenolic/stable/animal taint (data not shown). For this reason, the positive correlation highlighted between bitterness and off-flavour seems to support previous results [39], according to which bitterness was enhanced by animal aromas.

Overall, our findings suggest that during red wine tasting, odour–oral cross-modal interactions could modulate the perception of specific astringency sub-qualities and tastes. Specific olfactory characteristics such as spicy, dehydrated fruits and vegetal odours, could drive this modulation effect for complex, drying and unripe sub-qualities, and this should be further explored by specific experiments.

### 3.2. Olfactory Cues and Correlations between Sensory and Chemical Variables

In red wine astringency research, one of the biggest challenges is to find analytical methods able to predict the perceived astringency. Several studies investigated the correlation between astringency as a sensory parameter and measurements essentially based on compositional/metabolomic [68], spectrophotometric (e.g., 280 and 230 nm) [69], and precipitation techniques [70]. Thanks to these studies and to those investigating how other wine components (e.g., ethanol, pH, etc.) can influence astringency perception, our knowledge about this sensory stimulus has greatly expanded. However, most of these studies tested the correlation between chemicals and the overall astringency but did not pay attention to the different sub-qualities of this attribute. According to our recent results [40], and a few further studies addressing this subject [38,71], the current analytical methods are not able to predict astringency in all its sensory nuances, and their predictive power varies depending on the parameter/method applied.

We argue that odour–oral cross-modal interactions can affect the correlations between chemical and sensory parameters, thus interfering with the estimation of their predictive power. To test this hypothesis, we computed Pearson correlations between sensory (odour descriptors, astringency sub-qualities, and tastes) and chemical compositional parameters (total phenols, total proanthocyanidins, ethanol, reducing sugars, pH, titratable acidity, volatile acidity) across the 74 whole wines (WWs) and the corresponding deodorized wines (DWs). In this way, we were able to compare the correlations under two different tasting conditions: in the presence and in the absence of VOCs. This comparison is reported in Table 5 and Table 6, where several significant correlations (*p* < 0.05, *p* < 0.0001) were found. In most cases, the number of significant correlations and the magnitude of the correlation coefficients were higher for DWs than for WWs. In only a few cases, the magnitude of the correlation coefficients decreased, the direction of the sign switched, or the relationship got or lost its statistical significance. Among correlations between sub-qualities (Table 5), the unripe astringency was the only one showing a lower number of significant correlations and lower correlation coefficients in DWs compared to WWs. In the absence of olfactory cues, the unripe astringency is slightly negatively correlated with harsh and complex, while in the presence of odours, a weak negative relationship was detected also for drying, dynamic and particulate. The unripe mouthfeel was also significantly related to tastes. A good positive correlation with sourness was confirmed in the absence of VOCs, which seems coherent with the significant correlation with pH and titratable acidity. Moreover, in the absence of odours, the weak correlation with total proanthocyanidins and ethanol was lost and, among the considered sub-qualities, unripe became the only one not correlated with chemical parameters linked to polyphenols. These results support the idea that the unripe astringency is a multisensory feeling greatly impacted by VOCs perceptions.

As for unripe, also the complex sub-quality is defined as a mouthfeel including aroma sensations. However, unlike unripe, the magnitude of correlation coefficients with other sub-qualities increases in the absence of VOCs and the correlation with total phenols and proanthocyanidins became significant even if with low r values. Another result from this comparison refers to the particulate sub-quality. A higher number of significant correlations (from 3 to 8) with other sub-qualities, tastes and polyphenol parameters emerged in the absence of VOCs. This could suggest that odours can have a role in modulating the perception of this sensation; however, only low r values were computed.

Particulate and dynamic were never correlated to tastes in WWs, while in DWs they were both significantly correlated to bitter and, dynamic was also negatively correlated to sour. Unlike for WWs, for DWs all the seven astringency sub-qualities were significantly correlated to the bitter taste, with the largest correlation coefficients (WWs = 0.754; DWs = 0.785) confirmed between bitterness and harsh astringency.

Moving onto the correlations between chemical and sensory parameters, the magnitude of the correlation coefficients increased with wine deodorization. On the one hand, the absence of VOCs led to greater positive correlations between drying, harsh, dynamic sub-qualities and total polyphenols, total proanthocyanidins, ethanol and volatile acidity. On the other hand, the negative correlations of complex and surface smoothness with total phenols and proanthocyanidins were stronger for DWs. As an example, for DWs, total proanthocyanidins showed the greatest positive correlation coefficients with drying and dynamic, which increased from 0.571 to 0.703, and from 0.304 to 0.737, respectively, when compared to WWs. The correlations between volatile acidity and drying, harsh and dynamic became significant for DWs but not for WWs. All these results confirm previous findings on correlations between sensory and chemical parameters [38,40,71] and show the impact of cross-modal oral/olfactory sensory interactions on red wine perception.

The correlations that were detected in WWs between tastes and all the other sensory and chemical parameters (Table 6) were confirmed and reinforced in DWs. The only correlation that was not significant in WWs and became slightly significant in DWs was the one between reducing sugars and sweetness (from 0.099 to 0.595). This suggests that the overall aroma might modulate the perception of sweetness in red wine but further investigation is necessary. The significant positive correlation between pH and bitterness was stronger in DWs.

Among all the mentioned significant correlations, only a few can be considered good correlations (r > ±0.7). According to these, we can conclude that: bitterness and harsh astringency perceptions are strongly related independently from odour in-mouth multi-modal interactions; total proanthocyanidins is the better predictive chemical parameter for both drying and dynamic astringency, but the estimation of its predictive power is strongly affected by olfactory–oral cross-modal interactions.

To the best of our knowledge, this is the first time that this kind of comparison has been done. In our opinion, this approach, if applied to a wider variety of chemical parameters, could be helpful to research aimed at understanding which compounds and structures are related to different mouthfeel sensations. Results confirm the importance of cross-modal interactions on red wine perception and can help to optimize the current predictive analytical parameters/methods. Even if wine deodorization is time consuming, it offered interesting results and its further comparison with other approaches (e.g., nose clips) could represent an interesting future perspective. Only a few and recent reports focus on the impact of the odour stimuli on the perception of single sub-qualities rather than overall astringency, and no experiment was ever carried out on very diverse Italian red wines [40,72,73].

## Figures and Tables

**Table 1:** Standards and descriptors used to train the assessors in recognizing/distinguishing odours.

Reference Compounds	Concentration (µg/L) ^1^	Consensual Descriptors ^2^	Descriptors ^3^
2-phenylethanol	159.0	Floral, rose	Floral, rose, dried rose
Citral	76.8	Terpenic, citric, fruity	Sharp, lemon, sweet
linalool	14.3	Terpenic, floral	Citrus, floral, sweet, bois de rose, woody, green, blueberry
1-octen-3-one	1.7	Mushroom, earth, musk, vegetal	Herbal, mushroom, earthy, musty, dirty
cis-3-hexen-1-ol	157.5	Herbaceous, green, vegetal	Fresh, green, grassy, foliage, vegetable, herbal, oily
ethyl butyrate	27.7	Fruity	Fruity, juicy, fruity, pineapple, cognac
damascenone	14.4	Apple pie, baked apple	Natural, sweet, fruity, rose, plum, grape, raspberry, sugar
benzaldehyde	696.6	Bitter almond	Sharp, sweet, bitter almond, cherry
isoamyl acetate	10.4	Fruity, banana	Sweet, fruity, banana, solvent
gamma-dodecalactone	20.8	Dehydrated peach/apricot	Fatty, peach, sweet, metallic, fruity
Sotolone	2.0	Fenugreek, fennel, liquorice, nut, raisins	Sweet, caramellic, maple, sugar, burnt sugar, coffee
4-ethylguaiacol	118.2	Phenolic, smoked, woody	Spicy, smoky, bacon, phenolic, clove
4-ethylphenol	21	Phenolic, horse sweat	Phenolic, castoreum, smoky, guaiacol
Eucalyptol	30.1	Eucalyptol, balsamic	Eucalyptus, herbal, medicinal
Furaneol	7.0	Cotton candy, caramel, backed, toasted	Sweet, cotton candy, caramellic, strawberry, sugar,
ethyl caproate	35.4	Fruity, pineapple	Sweet, fruity, pineapple, waxy, green banana
Eugenol	30.9	Cloves, spicy	Sweet, spicy, clove, woody
Citronellol	48.0	Terpenic, floral	Floral, leathery, waxy, rose, citrus
phenylacetaldehyde	11.1	Honey, beeswax, fruity	Green, sweet, floral, hyacinth, clover, honey, cocoa
furfuryl acetate	79.7	Fruity, banana, sweet	Sweet, fruity, banana, horseradish
2,4,6-trichloroanisole	11.7	Cork taint	**-**
2-methyl-1-propanol	668	Amylic, chemical, grappa	Ethereal, winey
methanethiol	46.8	Garlic, sulfurous, vegetable	Sulfurous, onion, sweet, soup, vegetable
Ethanol	4.0 g/L	Alcoholic, ethereal, sharp	Strong, alcoholic, ethereal, medical

^1^ In distilled water; ^2^ Association frequency (percentage of judges that consensually associated the correct descriptor to a given standard solution) ≥ 85%; ^3^ The Good Scents Company.

**Table 2:** Three-way ANOVA (judges as random factor, grape variety and perception modality as fixed factors) computed to test the discrimination effect of in-mouth descriptors and to evaluate the impact of the perception modality (with and without odours, WWs and DWs, respectively) on oral sensory perception of the 74 red wines investigated.

Oral Descriptor	Model		Grape Variety		Perception Modality		Perception Modality*Grape Variety	
	F	*p*	F	*p*	F	*p*	F	*p*
Drying	15.488	**<0.0001**	14.557	**<0.0001**	0.191	0.662	1.438	0.157
Harsh	10.697	**<0.0001**	11.253	**<0.0001**	6.534	**0.011**	0.575	0.836
Unripe	11.541	**<0.0001**	6.744	**<0.0001**	11.293	**0.001**	2.046	**0.026**
Dynamic	10.241	**<0.0001**	16.396	**<0.0001**	11.001	**0.001**	1.976	**0.032**
Particulate/powder	5.858	**<0.0001**	1.064	0.387	2.567	0.109	0.891	0.541
Complex	12.593	**<0.0001**	3.658	**<0.0001**	54.233	**<0.0001**	1.368	0.189
Surface smoothness/velvet	7.881	**<0.0001**	10.517	**<0.0001**	4.313	**0.038**	0.807	0.622
Sweet	6.277	**<0.0001**	5.112	**<0.0001**	8.710	**0.003**	0.397	0.948
Sour	6.913	**<0.0001**	16.876	**<0.0001**	0.002	0.963	0.911	0.522
Bitter	7.915	**<0.0001**	10.126	**<0.0001**	13.342	**0.000**	1.149	0.321

In bold significant differences (Tukey, *p* < 0.05).

**Table 3:** Two-way ANOVA (judges as random factor and grape variety as fixed factor) computed to test the discrimination effect of olfactory descriptors among the 74 red wines investigated.

Olfactory Descriptor	Model		Grape Variety	
	F	*p*	F	*p*
Fruity	11.779	**<0.0001**	2.663	**0.003**
Dehydrated fruits	5.621	**<0.0001**	3.674	**<0.0001**
Dried fruits (nuts)	2.836	**<0.0001**	1.824	0.052
Floral	13.841	**<0.0001**	3.787	**<0.0001**
Vegetal	4.757	**<0.0001**	6.862	**<0.0001**
Spicy	6.549	**<0.0001**	2.478	**0.006**
Toasted	4.975	**<0.0001**	2.450	**0.007**
Woody	6.406	**<0.0001**	1.166	0.310
Earthy	1.903	0.006	1.679	0.081
Alcoholic	2.680	**<0.0001**	1.883	**0.044**
Off-flavour	5.766	**<0.0001**	4.508	**<0.0001**

In bold significant differences (Tukey, *p* < 0.05).

**Table 4:** Oenological parameters determined in the 74 single cultivar Italian red wines.

Parameter	Mean	Minimum	Maximum
Ethanol (% *v*/*v*)	13.89	11.42	16.62
Reducing sugars (g/L)	2.64	1.10	20.10
Titratable acidity (g tartaric acid/L)	5.75	3.99	9.99
pH	3.55	3.07	4.10
Total phenols (Folin-Ciocalteu) (mg (+)-catechin/L)	2354.46	703.59	5448.55
Proanthocyanidins (mg cyanidin chloride/L)	3364.80	627.75	6312.37

**Table 5:** Correlation coefficients (Pearson) between astringency and chemical variables. Comparison between WW and DW.

Variables	Drying		Harsh		Unripe		Dynamic		Complex		Surface Smoothness		Particulate	
	WW	DW	WW	DW	WW	DW	WW	DW	WW	DW	WW	DW	WW	DW
Drying	**1**	**1**	**0.391**	**0.440**	**−0.245**	0.001	**0.623**	**0.642**	**−0.278**	**−0.470**	**−0.651**	**−0.629**	0.060	**0.290**
Harsh	**0.391**	**0.440**	**1**	**1**	**−0.350**	**−0.230**	**0.285**	**0.590**	**−0.261**	**−0.344**	**−0.341**	**−0.406**	**0.253**	**0.260**
Unripe	**−0.245**	0.001	**−0.350**	**−0.230**	**1**	**1**	**−0.289**	−0.130	0.001	**−0.232**	0.044	−0.204	**−0.259**	−0.073
Dynamic	**0.623**	**0.642**	**0.285**	**0.590**	**−0.289**	−0.130	**1**	**1**	**−0.372**	**−0.501**	**−0.473**	**−0.465**	0.140	**0.238**
Complex	**−0.278**	**−0.470**	**−0.261**	**−0.344**	0.001	**−0.232**	**−0.372**	**−0.501**	**1**	**1**	**0.268**	**0.590**	−0.182	**−0.232**
Surface smoothness	**−0.651**	**−0.629**	**−0.341**	**−0.406**	0.044	−0.204	**−0.473**	**−0.465**	**0.268**	**0.590**	**1**	**1**	−0.155	**−0.376**
Particulate	0.060	**0.290**	**0.253**	**0.260**	**−0.259**	−0.073	0.140	**0.238**	−0.182	**−0.232**	−0.155	**−0.376**	**1**	**1**
Sweet	−0.056	−0.137	−0.077	−0.181	**−0.313**	**−0.353**	−0.114	−0.013	**0.355**	**0.252**	**0.289**	**0.293**	0.110	0.071
Sour	−0.197	−0.137	**−0.597**	**−0.526**	**0.538**	**0.597**	−0.043	**−0.284**	−0.095	−0.001	−0.009	−0.005	−0.173	−0.195
Bitter	**0.306**	**0.366**	**0.754**	**0.785**	**−0.237**	**−0.295**	0.130	**0.451**	−0.197	**−0.259**	−0.187	**−0.288**	0.080	**0.304**
Total phenols (Folin-Ciocalteu) (mg/L)	**0.469**	**0.622**	**0.284**	**0.506**	−0.189	0.166	**0.240**	**0.599**	−0.170	**−0.375**	**−0.292**	**−0.414**	**0.238**	**0.318**
Total proanthocyanidins(mg/L)	**0.561**	**0.703**	**0.297**	**0.577**	**−0.279**	0.110	**0.304**	**0.737**	−0.207	**−0.427**	**−0.304**	**−0.569**	0.163	**0.295**
Ethanol (% *v*/*v*)	**0.394**	**0.476**	**0.262**	**0.396**	**−0.264**	−0.137	0.094	**0.461**	0.016	−0.051	−0.178	−0.171	0.069	0.129
Reducing sugars (g/L)	−0.013	−0.014	−0.015	−0.165	0.059	0.055	−0.057	−0.017	0.206	0.125	0.043	0.196	0.109	−0.055
pH	−0.010	−0.010	**0.335**	**0.466**	**−0.274**	**−0.376**	−0.023	0.166	0.024	0.165	−0.071	0.106	0.134	0.055
TA (g tartaric acid/L)	0.084	0.163	**−0.248**	**−0.313**	**0.258**	**0.493**	0.080	−0.066	−0.041	−0.186	0.033	−0.197	−0.032	0.025
VA (g acetic acid/L)	0.193	**0.361**	0.201	**0.447**	−0.067	−0.158	0.215	**0.413**	−0.156	−0.198	−0.056	−0.165	0.051	0.086

In bold significant differences (Tukey, *p* < 0.05) (grey: *p* < 0.0001), whole wines (WWs), deodorized wines (DWs).

**Table 6:** Correlation coefficients (Pearson) between taste and chemical variables. Comparison between WW and DW.

Variables	Sweet		Sour		Bitter	
	WW	DW	WW	DW	WW	DW
Drying	−0.056	−0.137	−0.197	−0.137	**0.306**	**0.366**
Harsh	−0.077	−0.181	**−0.597**	**−0.526**	**0.754**	**0.785**
Unripe	**−0.313**	**−0.353**	**0.538**	**0.597**	**−0.237**	**−0.295**
Dynamic	−0.114	−0.013	−0.043	**−0.284**	0.130	**0.451**
Complex	**0.355**	**0.252**	−0.095	−0.001	−0.197	**−0.259**
Surface smoothness	**0.289**	**0.293**	−0.009	−0.005	−0.187	**−0.288**
Particulate	0.110	0.071	−0.173	−0.195	0.080	**0.304**
Sweet	**1**	**1**	**−0.277**	**−0.398**	**−0.243**	−0.131
Sour	**−0.277**	**−0.398**	**1**	**1**	**−0.668**	**−0.716**
Bitter	**−0.243**	−0.131	**−0.668**	**−0.716**	**1**	**1**
Total phenols (Folin-Ciocalteu) (mg/L)	−0.043	−0.118	−0.089	−0.179	0.168	**0.471**
Total proanthocyanidins (mg/L)	−0.067	−0.163	−0.102	−0.189	0.198	**0.498**
Ethanol (% *v/v*)	0.036	0.173	−0.210	**−0.331**	0.167	**0.327**
Reducing sugars (g/L)	0.099	**0.595**	−0.016	−0.079	0.019	−0.161
pH	−0.022	0.135	**−0.508**	**−0.656**	**0.371**	**0.529**
TA (g tartaric acid/L)	−0.058	−0.115	**0.459**	**0.621**	**−0.276**	**−0.424**
VA (g acetic acid/L)	−0.089	0.032	0.000	**−0.359**	0.145	**0.435**

In bold significant differences (Tukey, *p* < 0.05) (grey: *p* < 0.0001), whole wines (WWs), deodorized wines (DWs).

## Supplementary Materials

The following are available online at https://www.mdpi.com/2304-8158/9/11/1530/s1, Figure S1. SPME/GC-MS chromatograms (TIC) of a WW sample (a) compared to the corresponding DW (b).

## References

[1] Small D.M., Prescott J. (2005). Odor/taste integration and the perception of flavor. Exp. Brain Res..

[2] Prescott J. (2012). Chemosensory learning and flavour: Perception, preference and intake. Physiol. Behav..

[3] Noble A.C. (1996). Taste-aroma interactions. Trends Food Sci. Technol..

[4] Chironi S., Ingrassia M. (2013). Wine label design as a strategic tool to attract consumers: A marketing study on Sicilian wine positioning work. Riv. di Econ. Agrar..

[5] Vecchio R., Lisanti M.T., Caracciolo F., Cembalo L., Gambuti A., Moio L., Siani T., Marotta G., Nazzaro C., Piombino P. (2019). The role of production process and information on quality expectations and perceptions of sparkling wines. J. Sci. Food Agric..

[6] Peynaud E. (1987). The Taste of Wine: The Art and Science of Wine Appreciation.

[7] Charters S., Pettigrew S. (2007). The dimensions of wine quality. Food Qual. Prefer..

[8] Sáenz-Navajas M.P., Avizcuri J.M., Echávarri J.F., Ferreira V., Fernández-Zurbano P., Valentin D. (2016). Understanding quality judgements of red wines by experts: Effect of evaluation condition. Food Qual. Prefer..

[9] Li H. (2006). Wine Tasting.

[10] Guth H. (1997). Quantitation and sensory studies of character impact odorants of different white wine varieties. J. Agric. Food Chem..

[11] Bate-Smith E.C. (1954). Astringency in foods. Food Process. Packag..

[12] Chen J., Engelen L. (2012). Food Oral Processing: Fundamentals of Eating and Sensory Perception.

[13] Jiang Y., Gong N.N., Matsunami H. (2014). Astringency: A more stringent definition. Chem. Senses.

[14] Schöbel N., Radtke D., Kyereme J., Wollmann N., Cichy A., Obst K., Hatt H. (2014). Astringency is a trigeminal sensation that involves the activation of G protein-coupled signaling by phenolic compounds. Chem. Senses.

[15] Bate-Smith E.C. (1973). Haemanalysis of tannins, the concept of relative astringency. Phytochemistry.

[16] Kallithraka S., Bakker J., Clifford M.N. (1998). Evidence that salivary proteins are involved in astringency. J. Sens. Stud..

[17] Soares S., Vitorino R., Osório H., Fernandes A., Venâncio A., Mateus N., Amado F., de Freitas V. (2011). Reactivity of human salivary proteins families toward food polyphenols. J. Agric. Food Chem..

[18] Soares S., Brandão E., Mateus N., de Freitas V. (2017). Sensorial properties of red wine polyphenols: Astringency and bitterness. Crit. Rev. Food Sci. Nutr..

[19] Gawel R., Oberholster A., Francis I.L. (2000). A “Mouth-feel Wheel”: Terminology for communicating the mouth feel characteristics of red wine. Aust. J. Grape Wine Res..

[20] Perez-Jiménez M., Chaya C., Pozo-Bayón M.Á. (2019). Individual differences and effect of phenolic compounds in the immediate and prolonged in-mouth aroma release and retronasal aroma intensity during wine tasting. Food Chem..

[21] Fontoin H., Saucier C., Teissedre P.L., GLories Y. (2008). Effect of pH, ethanol and acidity on astringency and bitterness of grape seed tannin oligomers in model wine solution. Food Qual. Prefer..

[22] Watrelot A.A., Kuhl T.L., Waterhouse A.L. (2018). Friction forces of saliva and red wine on hydrophobic and hydrophilic surfaces. Food Res. Int..

[23] Hort J., Hollowood T.A. (2004). Controlled continuous flow delivery system for investigating taste–aroma interactions. J. Agric. Food Chem..

[24] Sáenz-Navajas M.P., Campo E., Avizcuri J.M., Valentin D., Fernández-Zurbano P., Ferreira V. (2012). Contribution of non-volatile and aroma fractions to in-mouth sensory properties of red wines: Wine reconstitution strategies and sensory sorting task. Anal. Chim. Acta.

[25] De la Fuente-Blanco A., Sáenz-Navajas M.P., Ferreira V. (2016). Levels of higher alcohols inducing aroma changes and modulating experts’ preferences in wine model solutions. Aust. J. Grape Wine Res..

[26] Cameleyre M., Lytra G., Barbe J.C. (2018). Static headspace analysis using low-pressure gas chromatography and mass spectrometry, application to determining multiple partition coefficients: A practical tool for understanding red wine fruity volatile perception and the sensory impact of higher alcohols. Anal. Chem..

[27] Sereni A., Osborne J., Tomasino E. (2016). Exploring retro-nasal aroma’s influence on mouthfeel perception of Chardonnay wines. Beverages.

[28] Niimi J., Eddy A.I., Overington A.R., Heenan S.P., Silcock P., Bremer P.J., Delahunty C.M. (2014). Aroma–taste interactions between a model cheese aroma and five basic tastes in solution. Food Qual. Prefer..

[29] Symoneaux R., Guichard H., Le Quéré J.M., Baron A., Chollet S. (2015). Could cider aroma modify cider mouthfeel properties?. Food Qual. Prefer..

[30] Labbe D., Damevin L., Vaccher C., Morgenegg C., Martin N. (2006). Modulation of perceived taste by olfaction in familiar and unfamiliar beverages. Food Qual. Prefer..

[31] Tournier C., Sulmont-Rosse C., Semone E., Issanchou S., Guichard E. (2009). A study on texture-taste-aroma interactions: Physico-chemical and cognitive mechanisms. Int. Dairy J..

[32] Caporale G., Policastro S., Monteleone E. (2004). Bitterness enhancement induced by cut grass odorant (cis-3-hexen-l-ol) in a model olive oil. Food Qual. Prefer..

[33] Saint-Eve A., Paci Kora E., Martin N. (2004). Impact of the olfactory quality and chemical complexity of the flavouring agent on the texture of low fat stirred yogurts assessed by three different sensory methodologies. Food Qual. Prefer..

[34] Poinot P., Arvisenet G., Ledauphin J., Gaillard J.L., Prost C. (2013). How can aroma–related cross–modal interactions be analysed? A review of current methodologies. Food Qual. Prefer..

[35] Sáenz-Navajas M.P., Campo E., Fernández-Zurbano P., Valentin D., Ferreira V. (2010). An assessment of the effects of wine volatiles on the perception of taste and astringency in wine. Food Chem..

[36] Sáenz-Navajas M.P., Arias I., Ferrero-del-Teso S., Fernández-Zurbano P., Escudero A., Ferreira V. (2018). Chemo-sensory approach for the identification of chemical compounds driving green character in red wines. Food Res. Int..

[37] Ferrer-Gallego R., Hernández-Hierro J.M., Rivas-Gonzalo J.C., Escribano-Bailón M.T. (2014). Sensory evaluation of bitterness and astringency sub-qualities of wine phenolic compounds: Synergistic effect and modulation by odours. Food Res. Int..

[38] Sáenz-Navajas M.P., Ferrero-del-Teso S., Jeffery D.W., Ferreira V., Fernández-Zurbano P. (2020). Effect of aroma perception on taste and mouthfeel dimensions of red wines: Correlation of sensory and chemical measurements. Food Res. Int..

[39] De-la-Fuente-Blanco A., Fernández-Zurbano P., Valentin D., Ferreira V., Sáenz-Navajas M.P. (2017). Cross-modal interactions and effects of the level of expertise on the perception of bitterness and astringency of red wine. Food Qual. Prefer..

[40] Piombino P., Pittari E., Gambuti A., Curioni A., Giacosa S., Mattivi F., Parpinello G.P., Rolle L., Ugliano M., Moio L. (2020). Preliminary sensory characterisation of the diverse astringency of single cultivar Italian red wines and correlation of sub-qualities with chemical composition. Aust. J. Grape Wine Res..

[41] Saenz-Navajas M.P., Campo E., Cullere L., Fernandez-Zurbano P., Valentin D., Ferreira V. (2010). Effects of the nonvolatile matrix on the aroma perception of wine. J. Agric. Food Chem..

[42] Muñoz-González C., Feron G., Guichard E., Rodríguez-Bencomo J.J., Martín-Álvarez P.J., Moreno-Arribas M.V., Pozo-Bayón M.A. (2014). Understanding the Role of Saliva in Odour Release from Wine by Using Static and Dynamic Headspace Conditions. J. Agric. Food Chem..

[43] Lytra G., Tempere S., de Revel G., Barbe J.C. (2012). Impact of Perceptive Interactions on Red Wine Fruity Aroma. J. Agric. Food Chem..

[44] Noble A.C., Arnold R.A., Buechsenstein J., Leach E.J., Schmidt J.O., Stern P.M. (1987). Modification of a standardised system of wine aroma terminology. Am. J. Enol. Vitic..

[45] ISO (1997). 3951. Sensory Analysis—Apparatus—Wine-Tasting Glass.

[46] ISO (2007). 8589. Sensory Analysis—General Guidance for the Design of Test Rooms.

[47] Rodríguez-Bencomo J.J., Muñoz-González C., Andújar-Ortiz I., Martín-Álvarez P.J., Moreno-Arribas M.V., Pozo-Bayón M.A. (2011). Assessment of the effect of the non-volatile wine matrix on the volatility of typical wine aroma compounds by headspace solid phase microextraction/gas chromatography analysis. J. Agric. Food Chem..

[48] ISO (2004). 4120. Sensory Analysis—Methodology—Triangle Test.

[49] Genovese A., Dimaggio R., Lisanti M.T., Piombino P., Moio L. (2005). Aroma composition of red wines by different extraction methods and gas chromatography SIM/mass spectrometry analysis. Ann. Chim..

[50] Piombino P., Genovese A., Gambuti A., Lamorte S.A., Lisanti M.T., Moio L. (2010). Effects of off-vine bunches shading and cryomaceration on free and glycosilated flavours of Malvasia delle Lipari wine. Int. J. Food Sci. Technol..

[51] Piombino P., Moio L., Genovese A. (2019). Orthonasal vs. retronasal: Studying how volatiles’ hydrophobicity and matrix composition modulate the release of wine odorants in simulated conditions. Food Res. Int..

[52] Organisation Internationale de la Vigne et du Vin (2015). Compendium of International Methods of Must and Wine Analysis.

[53] Singleton V.L., Orthofer R., Lamuela-Raventós R.M. (1999). Analysis of total phenols and other oxidation substrates and antioxidants by means of Folin-Ciocalteu reagent. Methods Enzymol..

[54] Di Stefano R., Cravero M.C., Gentilini N. (1989). Metodi per lo studio dei polifenoli dei vini. L’Enotecnico.

[55] Torchio F., Cagnasso E., Gerbi V., Rolle L. (2010). Mechanical properties, phenolic composition and extractability indices of Barbera grapes of different soluble solids contents from several growing areas. Anal. Chim. Acta.

[56] Vidal L., Antúnez L., Giménez A., Medina K., Boido E., Ares G. (2016). Dynamic characterization of red wine astringency: Case study with Uruguayan Tannat wines. Food Res. Int..

[57] Stalikas C.D. (2007). Extraction, separation, and detection methods for phenolic acids and flavonoids. J. Sep. Sci..

[58] Luo H., Schmid F., Grbin P.R., Jiranek V. (2010). Viability of common wine spoilage organisms after exposure to high power ultrasonics. Ultrason. Sonochem..

[59] García Martín J.F., Sun D.W. (2013). Ultrasound and electric fields as novel techniques for assisting the wine ageing process: The state-of-the-art research. Trends Food Sci. Technol..

[60] Liu L., Loira I., Morata A., Suárez-Lepe J.A., González M.C., Rauhut D. (2016). Shortening the ageing on lees process in wines by using ultrasound and microwave treatments both combined with stirring and abrasion techniques. Eur. Food Res. Technol..

[61] Zhang Q.A., Shen Y., Fan X.H., García-Martín J.F. (2016). Preliminary study of the effect of ultrasound on physicochemical properties of red wine. CyTA J. Food.

[62] Bonaldo F., Guella G., Mattivi F., Catorci D., Arapitsas P. (2020). Kinetic investigations of sulfite addition to flavanols. Sci. Rep..

[63] Celotti E., Stante S., Ferraretto P., Román T., Nicolini G., Natolino A. (2020). High Power Ultrasound Treatments of Red Young Wines: Effect on Anthocyanins and Phenolic Stability Indices. Foods.

[64] Slaghenaufi D., Ugliano M. (2018). Norisoprenoids, sesquiterpenes and terpenoids content of Valpolicella wines during aging: Investigating aroma potential in relationship to evolution of tobacco and balsamic aroma in aged wine. Front. Chem..

[65] Paronetto L., Dellaglio F. (2011). Amarone: A modern wine coming from an ancient production technology. Adv. Food Nutr. Res..

[66] Vidal L., Antúnez L., Giménez A., Medina K., Boido E., Ares G. (2017). Sensory characterization of the astringency of commercial Uruguayan Tannat wines. Food Res. Int..

[67] Vidal L., Giménez A., Medina K., Boido E., Ares G. (2015). How do consumers describe wine astringency?. Food Res. Int..

[68] Hufnagel J.C., Hofmann T. (2008). Quantitative reconstruction of the nonvolatile sensometabolome of a red wine. J. Agric. Food Chem..

[69] Boulet J.C., Trarieux C., Souquet J.M., Ducasse M.A., Caillé S., Samson A., Williams P., Doco T., Cheynier V. (2016). Models based on ultraviolet spectroscopy, polyphenols, oligosaccharides and polysaccharides for prediction of wine astringency. Food Chem..

[70] Ferrer-Gallego R., Rui G., Rivas-Gonzalo J.C., Escribano-Bailóna M.T. (2012). Interaction of phenolic compounds with bovine serum albumin (BSA) and α-amylase and their relationship to astringency perception. Food Chem..

[71] Vidal L., Antúnez L., Rodríguez-Haralambides A., Giménez A., Medina K., Boido E., Ares G. (2018). Relationship between astringency and phenolic composition of commercial Uruguayan Tannat wines: Application of boosted regression trees. Food Res. Int..

[72] Arapitsas P., Ugliano M., Marangon M., Piombino P., Rolle L., Gerbi V., Versari A., Mattivi F. (2020). Use of untargeted Liquid Chromatography–Mass Spectrometry metabolome to discriminate Italian monovarietal red wines, produced in their different terroirs. J. Agric. Food Chem..

[73] Parpinello G.P., Ricci A., Arapitsas P., Curioni A., Moio L., Segade S.R., Ugliano M., Versari A. (2019). Multivariate characterisation of Italian monovarietal red wines using MIR spectroscopy. OENO One.

