# Supplementary figures and images for: Exploring Olfactory–Oral Cross-Modal Interactions through Sensory and Chemical Characteristics of Italian Red Wines
Source: Foods. 2020 Oct 24;9(11):1530. doi: 10.3390/foods9111530 (PMC7692166; doi:10.3390/foods9111530)

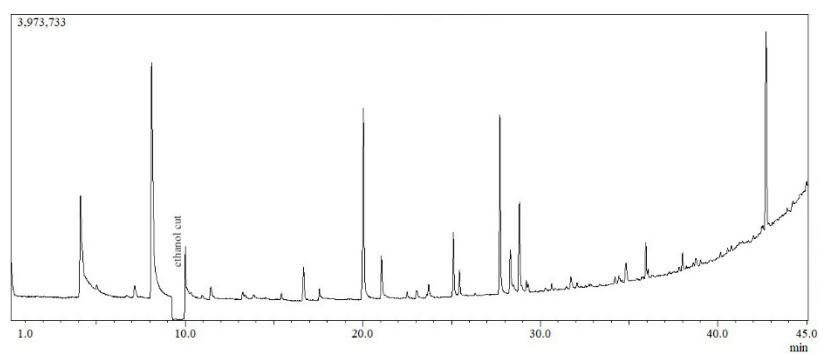

(a)

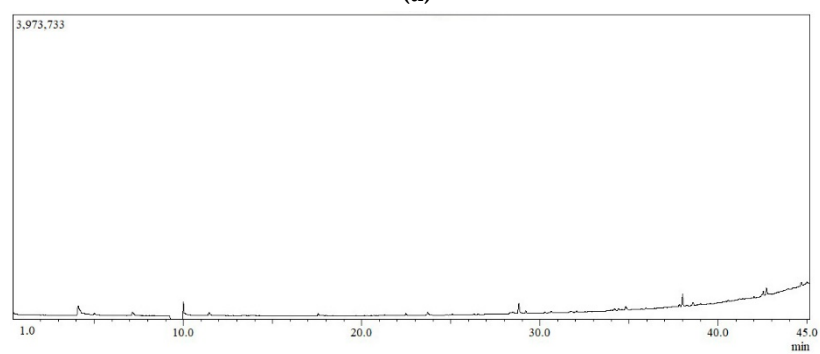

(b)

**Figure 1s.** SPME/GC-MS chromatograms (TIC) of a WW sample (a) compared to the corresponding DW (b).

Supplement: Supplementary file 1 [file foods-09-01530-s001.pdf]
